# Spatially Correlated Time Series and Ecological Niche Analysis of Cutaneous Leishmaniasis in Afghanistan

**DOI:** 10.3390/ijerph14030309

**Published:** 2017-03-17

**Authors:** Oyelola A. Adegboye, Majeed Adegboye

**Affiliations:** 1 Department of Mathematics, Physics and Statistics, Qatar University, 2713 Doha, Qatar; 2 Department of Information Technology, American University of Nigeria, 640001 Yola, Nigeria; dayomj@yahoo.com

**Keywords:** ecological niche model, environment, overdispersion, negative binomial, leishmaniasis, infectious disease

## Abstract

Leishmaniasis is the third most common vector-borne disease and a very important protozoan infection. Cutaneous leishmaniasis is one of the most common types of leishmaniasis infectious diseases with up to 1.2 million occurrences of new cases each year worldwide. A dynamic transmission multivariate time series model was applied to the data to account for overdispersion and evaluate the effects of three environmental layers as well as seasonality in the data. Furthermore, ecological niche modeling was used to study the geographically suitable conditions for cutaneous leishmaniasis using temperature, precipitation and altitude as environmental layers, together with the leishmaniasis presence data. A retrospective analysis of the cutaneous leishmaniasis spatial data in Afghanistan between 2003 and 2009 indicates a steady increase from 2003 to 2007, a small decrease in 2008, and then another increase in 2009. An upward trend and regularly repeating patterns of highs and lows were observed related to the months of the year, which suggests seasonality effect in the data. Two peaks were observed in the disease occurrence—January to March and September to December—which coincide with the cold period. Ecological niche modelling indicates that precipitation has the greatest contribution to the potential distribution of leishmaniasis.

## 1. Introduction

The World Health Organization estimates that there are 0.9–1.2 million annual cases of Leishmaniasis worldwide with approximately 0.2 to 0.4 million visceral leishmaniasis (VL) cases and 0.7 to 1.2 million cutaneous leishmaniasis (CL) cases each year [1]. Leishmaniasis is a vector-borne parasitic disease contracted through bites from infected female phlebotomine sand flies, which may result in mild self-healing cutaneous lesions to fatal visceral cases [2]. It is the third most common vector-borne disease and a very important protozoan infection [3]. The burden of the disease is overwhelming and the psychological effect can be disturbing. In some societies, women infected with this disease are stigmatized and may be deemed unsuitable for marriage and motherhood [4].

There are over 30 leishmania species known to infect humans [5]. The three main types of leishmaniasis disease are (i) visceral leishmaniasis (VL), often known as kala-azar and is the most serious form of the disease; (ii) cutaneous leishmaniasis (CL), which is the most common; and (iii) mucocutaneous [5]. Over 98 countries and territories are endemic for leishmaniasis with more than 70%–75% of the global CL cases occurring in ten countries: Afghanistan, Algeria, Brazil, Colombia, Costa Rica, Ethiopia, Iran, Peru, Sudan and Syria [1].

Afghanistan is a cutaneous leishmaniasis endemic country and the disease is of a serious health concern with about 200,000 estimated new cases of CL infection nationwide and 67,500 cases in Kabul alone [6]. There are two forms of cutaneous leishmaniasis (CL): zoonotic cutaneous leishmaniasis (ZCL) and anthroponotic cutaneous leishmaniasis (ACL). Most cases of leishmaniasis in Afghanistan are caused by *Leishmania tropica*, which is transmitted anthroponotically (i.e., humans are the reservoir) by the sand fly (*Phlebotomus sergenti*) [7,8,9]. On the other hand, ZCL is caused by *Leishmania major* with bites by *Phlebotomus papatasi* and occurs indigenously in rural northern Afghanistan [10]. However, in Afghanistan, public surveillance data are often reported without clear distinction between ZCL and ACL but are rather primarily focused on ACL [4,10,11].

A study of the risk factors for leishmaniasis incidence in Afghanistan found that household construction materials, design, density and presence of the disease in the neighborhoods are significant risk factors for ACL in Kabul [8]. Intervention measures such as simple window screens were demonstrated to reduce sand fly human vector contact [8]. Furthermore, evidence suggests that rodents may serve as natural hosts for CL and their infestations may increase the outbreak of the ZCL disease [11]. Similarly, the seasonality in the occurrence of ZCL in humans can be attributed to seasonal activity of the vector [11]. Many of the studies have not considered a spatial analysis approach to leishmaniasis in Afghanistan except for Adegboye [12]. For example, in Adegboye [12], a spatial hierarchical Bayesian model was used to analyze the spatial pattern of provincial level geo-referenced data of leishmaniasis in Afghanistan while Adegboye and Kotze [3] used random effects to assess spatial dependencies. They found excess risks of leishmaniasis in the northeastern and southeastern parts of Afghanistan [3,12].

The goal of this study is twofold—firstly, it is to explore the effect of time-varying environmental variables such as temperature, precipitation and elevation on the prediction of cutaneous leishmaniasis (CL) in Afghanistan. In surveillance counts data, underreporting or reporting delays of disease incidence are very common, which may give rise to overdispersion and blurred dependencies [13]. In this case, the usual Poisson model assumption of equal transmission incidence rates is doubtful; therefore, an alternative model that allows for possible overdispersion was used to achieve the first goal via negative binomial multivariate time series model. This model allows for overdispersion and seasonality. It is not unusual to discover this association to spread over a few time periods, and the association between environmental variables and disease occurrence over time may lead to bias unless the relationship is adequately modelled. We incorporated lag variables in the model to account for delay effects that spread over time.

The second goal of this study is to estimate the ecological niche and potential distribution of CL in Afghanistan using ecological niche modelling. It is not unusual to have some diseases more frequent in certain geographical areas, which could be attributed to environmental suitability of the region. Ecological niche modelling (ENM) provides a way to relate the geographical distributions of species to ecologic niches [14]. ENM is a growing area in spatial analysis of disease epidemiology [14,15], characterizing the distribution of the disease in a space defined by environmental parameters [15]. The use of ENM under the framework of geographic information system in spatial disease modelling cannot be overemphasized. For example, Du et al. [16] used ENM to identify the potential high risk areas for severe fever with thrombocytopenia syndrome (SFTS) in China. Adegboye and Kotze [15] used ENM to explore the geographical constraints that may favour the outbreak of the H5N1 virus in Nigeria. Similarly, ENM was used to estimate the current niche and potential distribution of mycetoma in Sudan and South Sudan [17], and to predict the zoonotic transmission niche of Ebola covering countries across Central and West Africa [18]. Recently, Chalghaf et al. [19] used ENM to study the effects of environmental variables on the geographical distribution of *P. papatasi* and to estimate environmental suitability index for CL in Tunisia.

The rest of the paper is structured as follows. Section 2 introduces the data set that motivated this study and the methods used. The results of the analyses are reported in Section 3. In Section 4, the concluding remarks will be presented.

## 2. Methods

### 2.1. Leishmaniasis Data

**Figure 1:**
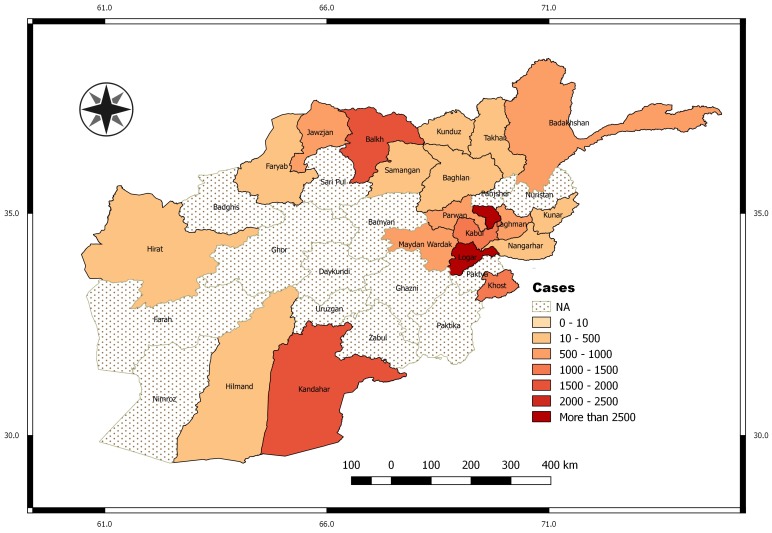
Map of Afghanistan showing aggregated incidence per 100,000 in all provinces in the study period (2003–2009). The **white** dotted areas indicate provinces without data.

Afghanistan is a landlocked country that is located in South Asia. The country is characterized by mountainous regions, and it is a leishmaniasis disease endemic country. Monthly data of cases of leishmaniasis reported to the Afghanistan Health Management Information System (HMIS) under the National Malaria and Leishmaniasis Control Programme (NMLCP) were obtained from the Ministry of Public Health (MoPH), Kabul, Afghanistan. The leishmaniasis infections were confirmed clinically or by examining the leishmania parasites in the skin lesion biopsy using calibrated ocular micrometre supported binocular light microscopy. There were 148,945 new cases of leishmaniasis recorded in a total of 20 provinces across Afghanistan between 2003 and 2009. Province level population denominators were obtained from the central statistics organization (CSO) of Afghanistan. Figure 1 shows the nationally aggregated monthly number of cases per 100,000 for the years 2003 to 2009.

### 2.2. Environmental Variables

Three environmental layers data sets were used in this study. The satellite-derived environmental-land surface temperature (LST) was obtained from the Moderate Resolution Imaging Spectroradiometer (MOD11 L2 version 6, USGS/Earth Resources Observation and Science (EROS) Center, Sioux Falls, South Dakota) at 1 km spatial resolution [20] while the elevation data was obtained from the Advanced Spaceborne Thermal Emission and Reflection Radiometer (ASTER) Global Emissivity Dataset (GED) version 3, USGS/Earth Resources Observation and Science (EROS) Center, Sioux Falls, South Dakota [21]. LST and elevation environmental layers were retrieved from the online data pool, courtesy of the National Aeronautics and Space Administration (NASA) Earth Observing System Data and Information System (EOSDIS) Land Processes Distributed Active Archive Center (LP DAAC), United States Geological Survey/Earth Resources Observation and Science (USGS/EROS) Center, Sioux Falls, South Dakota. The monthly accumulated rainfall data measured by the Tropical Rainfall Measuring Mission (TRMM: TMPA/3B43) [22] jointly conducted by NASA and the Japan Aerospace Exploration Agency (JAXA) was obtained from NASA.

### 2.3. Multivariate Time Series Analysis

Suppose $y_{i,t}$ is the monthly counts of leishmaniasis cases in province i=1,2,...,S at time series t=1,...,T. The disease counts are assumed to follow a negative binomial distribution, $y_{i,t}|y_{t-l}∼\mathrm{NegBin}\left(\mu_{i,t},\psi \right)$, with conditional mean

(1) $$\mu_{i,t}=\nu_{i,t}+\lambda_{it}y_{i,t-l}+\phi_{it}\sum_{j\neq i}w_{j,i}y_{j,t-l},$$

and conditional variance

$$\sigma_{i,t}^{2}=\mu_{i,t}+\psi \mu_{i,t}^{2},$$

where $y_{i,t-l}$ denotes the disease counts in province *i* at time t−l with lag l∈1,2,...; $\left(\phi_{it},\nu_{it},\lambda_{it}>0\right)$ and *ψ* is the overdispersion parameter. The parameters in $\mu_{i,t}$ is decomposed into two parts: $\log\left(\nu_{i,t}\right)=\log\left(n_{i,t}\right)+a_{i}+\alpha_{0}+\alpha_{1}t+\alpha_{2}\sin\left(\frac{2\pi }{12}t\right)+\alpha_{3}\cos\left(\frac{2\pi }{12}t\right)$ is the random "endemic" component that incorporates trend parameter, and sinusoidal wave of frequency to capture seasonality, and $n_{i,t}$ as the offset population size, whereas $\log\left(\lambda_{i,t}\right)=\beta_{0}+x_{i,t}\beta_{1}$ is the autoregressive component with explanatory variable(s) termed the "epidemic" component(s) [23]. The epidemic component is used to capture the occasional outbreaks while the endemic component explains the baseline rate of cases that is persistent with a stable temporal pattern [23].

In addition, $\log\left(\phi_{it}\right)=\gamma_{0}+c_{i}$ is the random neighbor-driven component that enables interdependency exploration between provinces with $w_{j,i}$ representing the spatial weight matrix (spatio-temporal component). The random effects a and c are assumed to be normally distributed with a mean 0 and variance Σ. Several models with varying structures and complexity were explored.

#### Model Selection

These models were validated using the proper scoring rules based on probabilistic one-step-ahead predictions to assess the best model. The smaller the score, the better the predictive quality [13]. A scoring rule S(P:y) measures the predictive quality of a stated predictive distribution *P* by comparing it with the actual observed value *y*. When the expected value under *P* becomes minimal given that the observed value *y* is indeed a realization from *P*, then we have a proper scoring rule. For this study, we shall use the logarithmic score LogS(P:y) and the ranked probability score RPS(P:y) for random effects model and Akaike information criterion (AIC) for models without random effects (see, for example, [13]).

These models are implemented in “R” [24] using the package “surveillance” (version 1.12.1) [25,26] as function “hhh4” [13], which can be downloaded from “R” website.

### 2.4. Ecological Niche Modelling

The main point of ecological niche modelling (ENM) is to relate the geographical distributions of species to ecologic niches [14]. The geographical variations in the species occurrences are often profoundly favoured by certain climatic and environmental constraints [14,15]. In ENM, the observed presence data (and pseudo-absence data) together with ecological variables at the sample region are used to provide a reasonable likelihood of the species being present at all other locations [15,27]. Such projections assume that a species distribution is mainly determined by its environmental requirements and not by other factors [27] (see, for example, [28] for more details on ENM).

MaxEnt version 3.3.3k [29] was used to estimate the ecological niches of leishmaniasis in Afghanistan via maximum entropy algorithm [30,31]. Although the MaxEnt software package [29] is particularly designed for species distribution/environmental niche modeling [32], the software can be easily adapted to disease niche modelling. The usual input of “species” presence locations will be replaced by the “disease” presence locations together with the set of environment variables. The analysis was carried out by dividing the occurrence data into 80% training data and 20% test data. The default convergence threshold of 0.00001 and 500 maximum iterations were used while 10 replicated models based on independent random partitions were used to get a robust model. The jackknife test of variable importance was used to estimate the relative contributions of the environmental variables to the model. Furthermore, the logistic output was used to estimate the suitability index (predicted probability of presence), which gives values between 0 and 1, indicating impossible conditions to highly suitable conditions.

The receiver operating curve (ROC) was used to investigate high predictive power of the model. A model with high predictive power will have a large area under the ROC curve (AUC), that is, the model accurately predicts the presence and absence. Following Hosmer and Lemeshow [33], a model with a AUC value between 0.7 and 0.8 is considered to provide acceptable discrimination while an AUC value above 0.8 is excellent (see [30,31,34] for further details on MaxEnt and [32] for an excellent practical guide to MaxEnt).

## 3. Results

### 3.1. Exploratory Data Analysis

**Figure 2:**
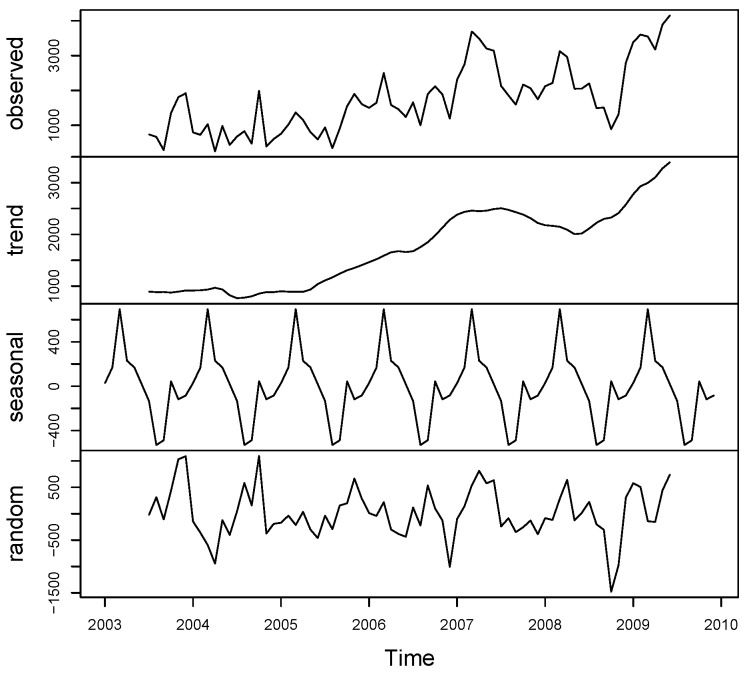
Decomposition of leishmaniasis time series into additive components. From the **top** panel to the **bottom** panel, time series plots of the disease, trend components, and seasonal components are indicated.

We begin the analysis by exploring the observed monthly cases of leishmaniasis in Afghanistan between 2003 and 2009. The top panel of Figure 2 shows the distribution of the monthly cases of the disease and a map of the aggregated monthly data across the seven years under study. The second panel is the trend component, which indicates a steady increase from 2003 to 2007, a small decrease in 2008, and then another increase in 2009. The third panel shows that the seasonal factor is the same for each year. An upward trend and regularly repeating patterns of highs and lows was observed related to the months of the year, which suggests seasonality in the data. The monthly profile indicates two peaks in the disease occurrence in Afghanistan between 2003 and 2009—January to March and September to December—which coincides with the cold period, while July is the hottest month and March is the wettest month. The largest seasonal factor is for March, and the smallest is for September, suggesting a peak in the cases of leishmaniasis in March and a trough in September of each year.

Similarly, the map in Figure 1 indicates higher disease incidence around the Kabul area (northeastern). Kabul City accounted for more than 50% of the total new cases in 2009, and this may be attributed to availability of health care facilities, which is crucial for data gathering in public health studies. The rate of infections is about the same for both male and female (around 50% each), while those in age group 4–14 years are more likely to be infected than other ages.

**Figure 3:**
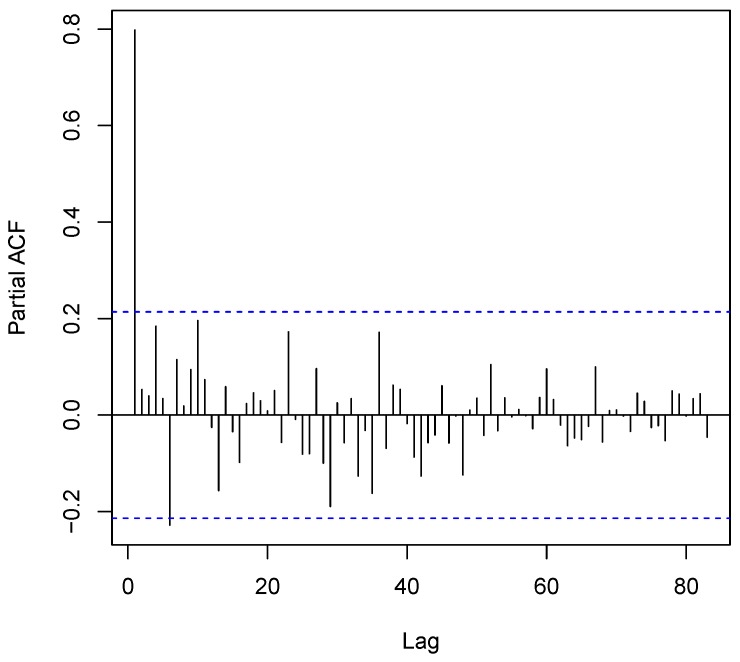
Autocorrelation plot of the “lag” (time span between observations) and the autocorrelation. The **blue** lines indicated 95% bounds for statistical significance.

The autocorrelation plot (Figure 3) shows a significant autocorrelation at lag 1 with ρ=0.7977 (D − W = 0.3719, *p*-value = 0). A further look using the partial autocorrelation function (PACF) shows a significant spike only at lag 1, which attests to the ACF. The autocorrelation at lag 1 is sufficient to explain the higher order autocorrelations, which implies that we can discard the longer lags.

### 3.2. Time Series Analyses

The model described in Section 2.3 has been fitted to the leishmaniasis count data to account for overdispersion and spatial dependency. The following model formulations were fitted to account for variability in the incidence of the disease as well as to investigate the effects of several risk factors:

**M1**  $\mu_{i,t}=\nu_{i,t}$; only seasonal variation in the endemic component, where $\log\left(\nu_{i,t}\right)=\log\left(n_{i,t}\right)+\alpha_{0}+\alpha_{1}t+\alpha_{2}\sin\left(\frac{2\pi }{12}t\right)+\alpha_{3}\cos\left(\frac{2\pi }{12}t\right)$
**M2**  $\mu_{i,t}=\nu_{i,t}+\lambda_{i,t}y_{i,t-1}+\phi_{i,t}\sum_{j\neq i}w_{j,i}y_{j,t-l}$; seasonal variation in the endemic component, autoregressive in the epidemic component, spatiotemporal component, but without adjusting for covariates in the epidemic component, where $\log\left(\nu_{i,t}\right)=\log\left(n_{i,t}\right)+\alpha_{0}+\alpha_{1}t+\alpha_{2}\sin\left(\frac{2\pi }{12}t\right)+\alpha_{3}\cos\left(\frac{2\pi }{12}t\right)$, $\lambda =\beta_{0}$ and $\log\left(\phi_{i,t}\right)=\gamma_{0}$, using the weights $w_{j,i}$=1/# neighbors of region *j* in the spatiotemporal component.
**M3**  $\mu_{i,t}=\nu_{i,t}+\lambda_{i,t}y_{i,t-1}+\phi_{i,t}\sum_{j\neq i}w_{j,i}y_{j,t-l}$; seasonal variation in the endemic component with random effects, autoregressive in the epidemic component with covariate adjustment and spatiotemporal component with random effects,

where $\log\left(\nu_{i,t}\right)=\log\left(n_{i,t}\right)+a_{i}+\alpha_{0}+\alpha_{1}t+\alpha_{2}\sin\left(\frac{2\pi }{12}t\right)+\alpha_{3}\cos\left(\frac{2\pi }{12}t\right)$, $\lambda =\beta_{0}+\beta_{1}*\log ALT+\beta_{2}*Temperature+\beta_{3}*Precipitation+\beta_{4}*Wind$, $\log\left(\phi_{i,t}\right)=\gamma_{0}+c_{i}$. The weights $w_{j,i}$=1/# neighbors of region *j* in the spatiotemporal component.

Following the results from the exploratory analysis (See Figure 2), we fitted a parameter driven Model M1 with only fixed parameters, including only a trend parameter and sinusoidal wave of frequency to capture seasonality in the ’endemic’ part of Equation (1). Table 1 displays the results from our model applied to the leishmaniasis data. Model M1 was fitted as a baseline model to assess the appropriateness of negative binomial model rather than a Poisson model. Model M1 provides a better fit (AIC = 133, 03.79) when compared with the Poisson model with an AIC of 404,954.22. The negative binomial baseline model has a significant overdispersion parameter, ψ=7.596 (95% Confidence Interval (CI) 7.027–8.166), implying evidence for residual overdispersion.

It is not unusual to discover this association to spread over a few time periods. Leishmaniasis incidence for any given month was autoregressed on the previous month’s infections. From exploratory analysis, one month lag autoregression was found to have a significant contribution. Additional parameters were introduced in the second Model M2. One month lag was included in the ’epidemic’ autoregressive effect and spatiotemporal component. The inclusion of these two terms improve the model via smaller AIC, LogS and RPS as shown in Table 1. Model (M2) provides an improvement over M1 with an AIC of 12,815.86 and overdispersion parameter ψ=5.204 (95% CI 4.789–5.617). Finally, in Model M3, in addition to adjusting for covariates (Altitude, Precipitation, Temperature and Wind), each region gets its own fixed parameter in the endemic component as well as random neighbour-driven components that enable interdependency exploration between region.

**Figure 4:**
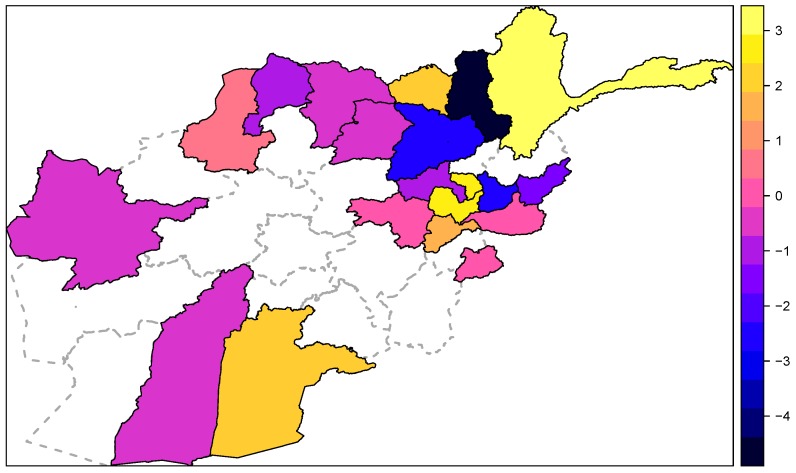
Map of random intercepts in the endemic component. The colour indicates the level of endemicity in each provinces after adjusting for variables such population and environmental layers. The bi-chromatic range from **dark blue** to **yellow** indicates low relative endemic incidence to high endemic incidence.

The predictive capabilities of the models were assessed through one-step-ahead predictions of the last five months. Model M3 was chosen as the best model based on smaller RPS and LogS among the three models. The interpretation of the parameter values are on the log scale, for example, in M3 $e^{\alpha_{1}}$ = exp(Trend) = 1.063 represents the seasonality-adjusted factor by which the basic endemic incidence increases per month. The estimates for trend and the seasonal components in the three models are quite similar and significant overdispersion parameter. The map in Figure 4 displays estimated spatially correlated random effects. It revealed that three provinces—Takhar, Baghlan and Laghman—show a low endemic incidence of CL in Afghanistan using variables from Model M3. The results of the best model confirmed the significant effect of precipitation and temperature. Likewise, the trend and the sinusoidal wave of frequency exerted a significant effect on leishmaniasis. However, no significant relationship of wind was detected as shown in Table 1.

**Figure 5:**
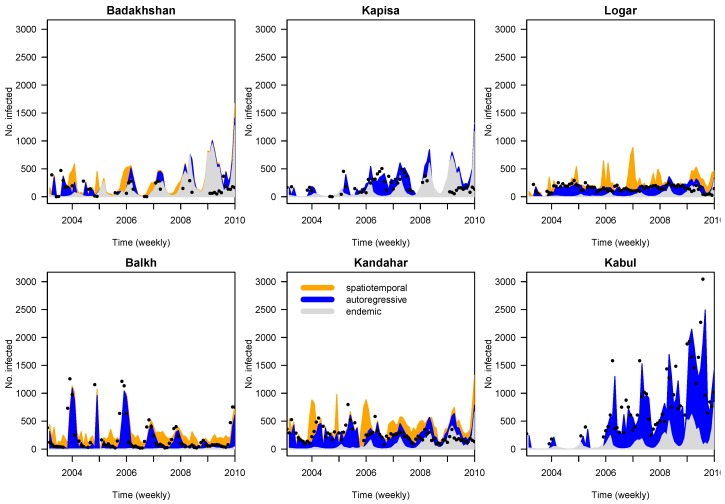
Plots of predicted values for cutaneous leishmaniasis in six selected provinces in Afghanistan showing the relative contributions of the three components (spatiotemporal, autoregressive and endemic) in Model 3. The dots represent the observed disease counts.

The fitted values for six provinces with the highest Leishmaniasis incidence in Afghanistan from Model 3 were presented in Figure 5. The plot is split into three components: the spatiotemporal, the autoregressive and the endemic components.

### 3.3. Suitability Index and Relative Importance of Environmental Layers for the Occurrences of Leishmaniasis

**Figure 6:**
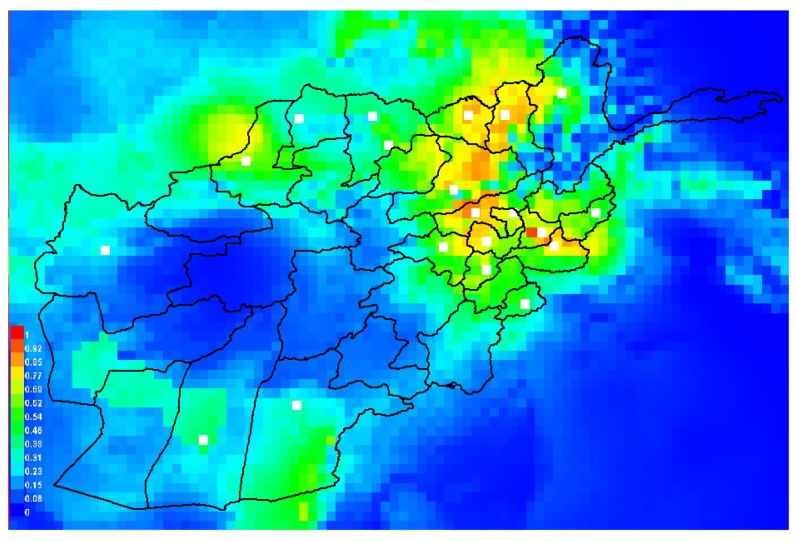
The ecological niche modelling of leishmaniasis in Afghanistan using MaxEnt: Predicted occurrence probability map of cutaneous leishmaniasis in Afghanistan bases on outbreak data with environmental variables in Table 2. Dots represent the administrative headquarters points used for the ENM. Warmer colors in the habitat suitability map show areas with better predicted conditions.

Figure 6 presents the map of the suitability habitat for leishmaniasis in Afghanistan estimated using MaxEnt ecological niche modeling. The eastern and northeastern regions of the country are potential high risk areas for leishmaniasis represented by warmer colors in Figure 6. These areas represent previously known occurrences of leishmaniasis. The relative contribution of each environmental layers to the final training MaxEnt model using the average of the 10 replicates is presented in Table 2. Similarly, in Table 2, the results of the jackknife test of variable importance were presented in terms of ranking of training model gains when using each variable separately. The environmental layer with highest gain when used in isolation is the afg-prec4, which appears to have the most useful information by itself. The environmental layer that decreases the gain the most when it is omitted is afg-prec4, which appears to have the most information that is not present in the other variables. The nine environmental layers shown in Table 2 most favour the occurrence of leishmaniasis. The mean AUC value for the training models was 0.929, and that of the testing model was 0.756, which is an indication of good performance.

## 4. Discussion

The impact of environmental variables on leishmaniasis cannot be ruled out and human activities play a significant role in the dispersion of the vectors, thereby changing the geographical distribution of the disease. Previous studies have investigated the effects of environmental variables on the occurrence of leishmaniasis [19,35,36,37,38,39,40,41]. Several authors have previously used times series analyses to study infectious disease incidences [13,42,43,44,45,46,47,48]. These approaches were used to investigate the dynamic pattern of the disease over different temporal and spatial scales. Geospatial and spatio-temporal techniques as well as ecological niche analysis are very common approaches to study the association between environmental layers and leishmaniasis (see, for example, [38,49,50,51]).

In this study, the dependent variable is the monthly time series of leishmaniasis in the provinces of Afghanistan between 2003 and 2009. The explanatory variables are the environmental layers: precipitation, temperature and altitude. Although cutaneous leishmaniasis is a worldwide health issue [52], Afghanistan, Algeria, Brazil, Colombia, Iran (Islamic Republic of) and the Syrian Arab Republic accounted for over 95% of the cases. There are several challenges in studying the effect of environmental factors on the transmission of infectious diseases in Afghanistan due to high variation in the landscape and climate. In the same vein, the association between environmental layers and disease occurrence over time may lead to bias unless the relationship is adequately modelled. When the association between disease incidence and environmental layers spans into the future, modelling techniques that incorporate autoregressive components is deemed necessary.

Spatial time series analysis was used to investigate the effect of time-varying environmental layers on the occurrence of cutaneous leishmaniasis in Afghanistan by using a flexible NegBin model that allows for overdispersion and varying components’ specification. In this model, different types of variation and correlation were incorporated within a single model. Our results show two significant peaks—January to March and September to December—with the highest peak in March, suggesting a peak in the cases of leishmaniasis in March and a trough in September of each year. It is probable that the seasonality observed in the cases of leishmaniasis could be attributed to the abundance of sandflies (vector carrying the leishmania parasite), which vary between species, location and year of collection [53]. In the Mediterranean context, two peaks of species *P. perniciosus* were recorded in July and September in Tunis, Almeria, Algarve and Catania [53]. A tri-modal peak of *P. tobbi* was recorded in Cyprus, and a bi-modal peak was observed in Turkey, while a single peak of *P. ariasi* was observed in France and Georgia [53]. Gálvez et al. [39] reported that the seasonal abundance of *P. perniciosus* sandflies in Central Spain had two peaks in July and September, while it reported a single peak of *P. ariasi* in August. El-Shazly et al. [40] reported peaks of *P. perniciosus* in July and October in Egypt.

Rodents may serve as natural hosts for cutaneous leishmaniasis [11]. The seasonality in the occurrence of zoonotic cutaneous leishmaniasis in humans was attributed to seasonal activity of the rodents, which are natural reservoir hosts of the disease [11,54]. Regions with good vegetation for plant growth have been described to provide shelter and food for rodents, thereby providing ideal sand fly habitats [55].

Previous studies have described the occurrence of the disease as seasonal [38,41,55]. The seasonality in our data was also confirmed by the significance of trend and sinusoidal wave term as well as precipitation and temperature in our final model. These findings are similar to other studies, indicating a positive association between precipitation, temperature and leishmaniasis [41,55].

Furthermore, we explored the environmental constraints that favoured the occurrences of leishmaniasis via ecological niche models (ENM) to predict suitable environmental conditions that favour the transmission of leishmaniasis in Afghanistan. We used the ENM technique to predict the areas of potential high risk for leishmaniasis disease using maximum entropy modelling (MaxEnt model) [30,31,34]. Previous authors have used ENM to estimate environmental suitability index for leishmaniasis [19,49]; however, to the best of our knowledge, this the first study that provides a suitability index for leishmaniasis in Afghanistan.

The ENM estimates indicate that the eastern and northeastern regions are potential high risk areas for leishmaniasis in Afghanistan. Precipitation and temperature provide significant contribution in predicting the suitable condition for leishmaniasis, with average precipitation in April contributing the greatest. This is similar to studies that found precipitation to contribute significantly to the predictive probability of the presence of leishmaniasis [19,41] and sandflies [55].

## 5. Conclusions

The impact of environmental influences on leishmaniasis cannot be ruled out and human activities play a significant role in the dispersion of the vectors, thereby changing the geographical distribution of the disease. In our study, precipitation in the month of April provided the greatest contribution to the ecological niche of leishmaniasis in Afghanistan. The use of geographical information system and remotely sensed satellite derived environmental layers to infectious disease modelling have been used in this study. The major limitation of this study is the use of aggregated leishmaniasis surveillance data in which we were unable to distinguish between ACL and ZCL entities. Actually, ACL lesions have long incubation and chronic persistence, whereas ZCL lesions are characterized by shorter incubation and rapid healing, which may have influence on seasonality of diagnosis. Moreover, the disease incidences are calculated from passive case detection data, and, therefore, the spatial distribution of the disease could be significantly influenced by the distribution of public health services. This study is solely based on the ecological aspect of disease transmission. Our results could be validated by incorporating sandfly and rodent distribution data. However, findings in this study serve as a versatile tool for studying and understanding transmission and spread of leishmaniasis. The model may be useful in different frontiers of the disease upsurge and possibility of its containment or eradication.

## Figures and Tables

**Table 1:** Parameter estimates (95% ${}^{‡}$CI) from the five models fitted to the leishmaniasis data.

Parameter	M1	M2	M3
Endemic component			
Intercept ($\alpha_{0}$)	6.940 (6.660, 7.219)	5.978 (5.701, 6.255)	
Trend ($\alpha_{1}$)	0.031 (0.024, 0.036)	0.003 (−0.004, 0.011)	0.061 (0.049, 0.072)
Sine ($\alpha_{2}$)	0.349 (0.004, 0.374)	1.139 (−0.050, 0.356)	1.567 (1.026, 1.601)
Cosine $\alpha_{3}$	0.997 (0.101, 0.485)	1.436 (0.866, 1.392)	0.577 (0.567, 1.142)
IID Random term *a*			2.357 (1.244, 3.471)
Epidemic Autoregressive component			
Intercept $\beta_{0}$		−0.065 (−0248, 0.122)	0.250 (1.575, 2.074)
LogALT $\beta_{1}$			0.064 (0.030, 0.175)
Precipitation $\beta_{2}$			0.011 (0.007, 0.017)
Temperature $\beta_{3}$			0.050 (0.021, 0.089)
Wind $\beta_{4}$			0.003 (−0.052, 0.057)
Spatiotemporal			
Spatiotemporal $\gamma_{0}$		−2.20 (−3.219, 3.820)	−5.003 (−7.037, −2.969)
Overdispersion			
Overdispersion *ψ*	7.597 (7.027, 8.166)	5.204 (4.789, 5.617)	3.284 (3.007, 3.561)
AIC	13,303.79	12,815.86	
LogS	6.700	6.348	6.178
RPS	120.128	93.568	90.204

${}^{‡}$CI: Confidence Interval.

**Table 2:** Estimates of relative contributions of the environmental variables to the Maxent model.

Variable	Description	Percent Contribution	Jackknife Rank
afg-prec4	Mean precipitation for April	54.5	1
afg-prec9	Mean precipitation for September	15.3	9
afg-tmean1	Mean temperature for January	9.9	2
afg-tmean7	Mean temperature for July	7.1	11
afg-prec10	Mean precipitation for October	5.6	7
afg-prec6	Mean precipitation for June	2.1	10
afg-prec7	Mean precipitation for July	1.7	4
afg-prec8	Mean precipitation for August	1.4	8
afg-prec12	Mean precipitation for December	1.2	5
afg-prec2	Mean precipitation for February	0.6	3
afg-prec11	Mean precipitation for November	0.6	6
